# Dietary Carnitine and Carnosine Increase Body Lean in Healthy Cats in a Preliminary Study

**DOI:** 10.3390/biology10040299

**Published:** 2021-04-05

**Authors:** Kiran S. Panickar, Mary C. DeBey, Dennis E. Jewell

**Affiliations:** 1 Hill’s Pet Nutrition Inc., Topeka, KS 66617, USA; kiran_panickar@hillspet.com (K.S.P.); mary_debey@hillspet.com (M.C.D.); 2 Department of Grain Science and Industry, Kansas State University, Manhattan, KS 66506, USA

**Keywords:** carnitine, carnosine, body lean, metabolomics, cytokines

## Abstract

**Simple Summary:**

Cats, like mammals in general, experience lean body mass loss in later life. This study shows that two dietary interventions offset that loss: L-carnitine and carnosine. The combination did not change body lean. Interestingly, the combination resulted in an increased circulating concentration of 8 of the 10 cytokines measured, while L-carnitine alone resulted in decreased concentrations. Thus, L-carnitine could benefit the healthy cat while in some disease states it may be beneficial to increase both L-carnitine and carnosine.

**Abstract:**

The need to maintain body lean as cats age is shown in both health and disease. In healthy cats, body lean is associated with enhanced movement and overall longevity. In many disease states (i.e., renal disease, obesity), an enhanced or minimally maximal support of body lean is associated with quality of life and is a nutritional goal in aiding in the management of the disease. This study was designed to investigate the effect of these two dietary components and their combination on body composition and circulating factors of health, including metabolomics analysis and cytokine concentration. The foods that were fed for 169 days to four groups of cats and consisted of control food (formulated to meet the nutritional needs of all adult cats), carnitine-enhanced food (control food plus 300 mg/kg L-carnitine), carnosine-enhanced food (control food plus 1000 mg/kg carnosine), and food enhanced with both (control plus 300 mg/kg carnitine and 1000 mg/kg carnosine). Dietary enhancement with L-carnitine and carnosine increased body lean at the end of the study compared to the cats consuming the control food or the combination food. The cats consuming L-carnitine alone had a decreased concentration of circulating cytokines, while those consuming the combination food had an increased concentration of glucose, pyruvate, succinate, and circulating cytokines.

## 1. Introduction

Increasing muscle carnitine and carnosine in healthy adult felines can potentially increase muscle metabolism, increase energy expenditure, and improve body composition over a prolonged period. Carnitine may be synthesized in the liver from lysine and methionine and is found in feline foods that incorporate striated muscle. From a cellular perspective, carnitine facilitates the transport of fatty acids into the mitochondria, which helps generate metabolic energy. Carnitine biology has been exhaustively reviewed [1,2,3]. Briefly, L-carnitine is involved in the transport of fatty acids across the mitochondrial membrane into the mitochondrial matrix where they undergo β-oxidation to acetyl CoA. Acetyl CoA then enters the citric acid cycle for complete oxidation. In simple terms, L-carnitine is needed by the cells to transport fatty acids into the mitochondria where they are metabolized to create energy. High concentrations of carnitine are found in striated muscle, and muscle concentration varies by breed and species [4,5].

Carnosine is a dipeptide composed of β-alanine and L-histidine [6] that is synthesized in the skeletal muscle. Muscle carnosine may decline with age [7]. However, supplementation with carnosine increased muscle carnosine levels [8]. The pH buffering effect and the antioxidant effects of carnosine have been well-established. Due to its predominance in the skeletal muscle, carnosine or β-alanine have also been hypothesized to have an ergogenic effect (enhance performance in high-energy activities) in human studies [9,10,11]. In humans, several studies have reported that β-alanine supplementation can increase high-intensity intermittent exercise performance and/or training adaptations. Improved cognitive performance with carnosine supplementation has also been demonstrated in human studies [12,13].

In cell cultures, carnosine upregulated the mitochondrial respiratory and cellular ATP content of astrocyte cultures in the recovery period following an ischemic injury (oxygen-glucose deprivation) [14]. The health benefits of carnosine, in particular its effects on energy metabolism, have been demonstrated [15]. To our knowledge, reports of carnosine supplementation in cat food and their subsequent effects on body composition and/or circulating levels of inflammatory markers has not been reported. The nutritional requirements of cats do not include carnitine or carnosine as essential nutrients.

Therefore, this study was designed to evaluate if carnitine or carnosine alone, or a combination could be beneficial in improving lean body mass in felines and their effects on circulating cytokines as markers of systemic low-grade inflammation.

## 2. Materials and Methods

### 2.1. Animals and Housing

The study was conducted at the Science and Technology Center of Hills Pet Nutrition Center, Topeka, KS. All study protocols were reviewed and approved by the Institutional Animal Care and Use Committee, Hill’s Pet Nutrition, Topeka, KS, USA (Permit Number: CP09–478) and complied with the National Institutes of Health guide for the care and use of Laboratory animals (NIH Publications, No. 8023, revised 1978). All cats were housed in groups of 10–12 in a room with natural light; access to porches; toys; and multiple levels, and were maintained in the same group housing throughout the study. While access to water was ad libitum, each cat was offered a specified amount of food daily by the use of individual electronic feeders that identified each cat by its microchip and monitored the amount of food it consumed. The allotted daily portion of food was available beginning in the morning, and cats had unlimited access to the electronic feeders throughout the day.

### 2.2. Group Assignments and Study Design

**Figure 1:**
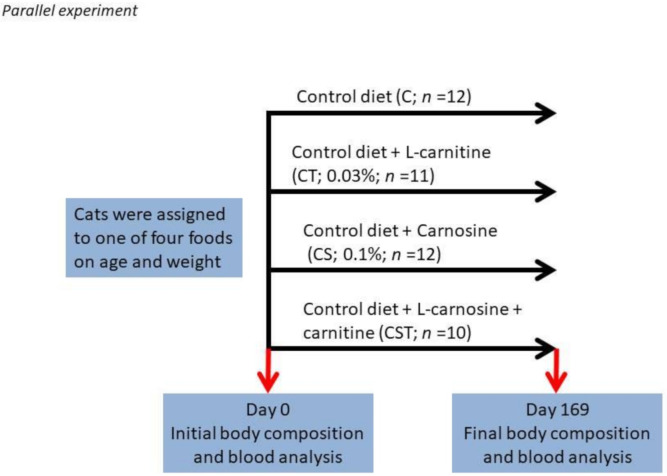
Feeding experimental design.

A total of forty-five adult cats were included in the study. None had chronic systemic disease on the basis of results of physical examination, complete blood count determination, serum biochemical analyses, urinalysis, and fecal examination for parasites. Age of the cats ranged from 6.2 to 13.9 years (and was not different between treatments). The base diet was an adult cat food formulated to meet the Association of American Feed Control Officials (AAFCO) requirements of adult cats. Cats were assigned to one of four groups by body weight and age as shown in Figure 1. The duration of the study was approximately 24 weeks, and each group was fed a single diet for the entire course of the study. Study foods are reported in Supplementary Table (Table S1). Blood was collected to obtain plasma at the beginning (Day 0) and end (Day 169) of the study.

### 2.3. Body Composition Measurement

Body mass and composition were assessed by dual-energy X-ray absorptiometry (DXA-QDR-4500, Hologic) scan analysis at the beginning, and end-of-the-study weight was reported in grams.

### 2.4. Complete Blood Count (CBC) and Blood Chemistry

Blood chemistries were measured using a Cobas 6000 c501 analyzer. CBC’s were analyzed on a Sysmex xt2000i instrument. All blood parameters were measured on days 1 and 169.

### 2.5. Cytokines

Cytokines were measured by ELISA according to kit instructions (EMD Millipore, Chicago, IL). Serum levels of cytokines assessed included Flt-3L (FMS-like tyrosine kinase 3), IFN-γ, TNF-α, IL-4, IL-6, IL-8, IL-12, IL-13, IL-18, and MCP-1 (monocyte chemotactic protein 1 or CCL2). The unit of measurement was pg/mL. The ratio of the change in cytokine concentration was calculated by subtracting the log of the initial concentration from the log of the final concentration. Analysis was completed on this log transformed data.

### 2.6. Plasma Metabolomics

Plasma metabolites were measured by a commercial laboratory (Metabolon; Durham, NC). Extracted supernatant was split and run on gas chromatography and liquid chromatography mass spectrometer platforms in a randomized order. Gas chromatography (for hydrophobic molecules) and liquid chromatography (for hydrophilic molecules) were used to identify and provide relative quantification of small metabolites present in plasma samples. Endogenous biochemicals included amino acids, peptides, carbohydrates, lipids, nucleotides, cofactors, and vitamins. Data for each individual compound were normalized by calculating the median values for each run-day block (block normalization). This minimized any inter-day instrument gain drift but did not interfere with intra-day sample variability. Specific analytes are reported when there was a difference in the final concentration of a metabolite in a test group when compared to the control-fed cats at the end of the study and there was no difference in concentration of that analyte initially.

### 2.7. Statistical Analysis

**Figure 2:**
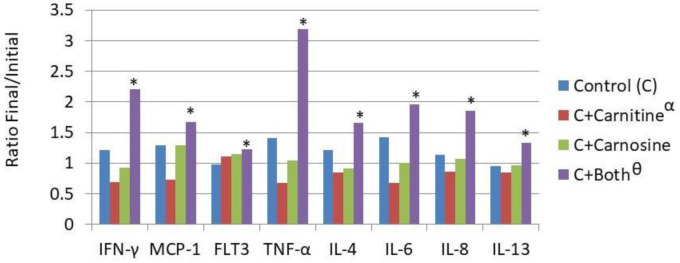
Dietary influence on circulating cytokine concentration. IFN-γ—Intrerferon gamma; MCP-1—Monocyte Chemoattractant Protein-1; Flt-3L—Fms-like tyrosine kinase 3 ligand; TNFα—Tissue Necrosis Factor α; IL-4—Interleukin 4; IL-6—Interleukin 6; IL-8—Interleukin 8; IL-13—Interleukin 13; € Treatments are as follows: Control (C)—a traditional adult cat food; C+Carnitine—control food supplemented with 300 mg/kg L-carnitine; C+Carnosine—control food supplemented with 1000 mg/kg carnosine; * Means are increased from initial values (*p* < 0.05); ^Θ^ The carnitine + carnosine treatment increased cytokine concentration (*p* < 0.05); ^α^ The carnitine treatment reduced cytokine concentration (*p* < 0.05).

Body composition and blood samples reported in Table 1 and Table 2 were evaluated using PROC mixed with SAS (SAS 9.4) with time, carnitine, carnosine, and their interaction as main effects. Post hoc analysis was completed using the PDIFF command (SAS 9.4). For the cytokine analysis (Figure 2), multivariate analysis was completed using the MANOVA option of PROC GLM (SAS 9.4), and to test if the ratio of the difference of the log of the initial concentration subtracted from the log of the final concentration was different than 1. Class variables for the multivariate analysis were cytokine, carnitine, carnosine, and animal ID. For the individual cytokine analysis, carnitine, carnosine, and their interaction were used as class variables. Metabolomics data (Table 3 and Table 4) were statistically analyzed by Metabolon, Inc. Briefly, for metabolomics, Welch’s two-sample t-tests and matched pairs t-test were used to analyze the data. The statistical analyses for metabolomics were performed on natural log-transformed data. A *p* value of *p* < 0.05 was used as a significance cutoff.

## 3. Results

### 3.1. Body Composition

There was no significant difference in lean mass, fat mass, or body weight between groups of cats at the beginning of the study. By Day 169, at the end of the study, there were significant differences noted between groups. The cats eating foods supplemented with carnitine only (CT) or carnosine only (CS) gained more body lean mass than cats eating control food (C) or food supplemented with both carnitine and carnosine (CST). There was no significant difference in body lean of cats fed C compared to cats fed CST. Cats eating CT food had a greater weight at the end of the study as compared to all other groups. This is because the lean gain in the CS cats was offset by fat loss that was not present in the CT group. Caloric intake was similar among groups (Table 1).

### 3.2. Complete Blood Count (CBC) and Blood Chemistry

Laboratory measurements of CBC and chemistry values were within normal parameters for the cats throughout the study. Mean glucose concentration in this study was 98–115 mg/dL (normal limits 67–157 mg/dL). The cats eating CST had an increased ending glucose concentration as compared to all other treatment groups. Mean triglycerides were 28–44 mg/dL (normal limits 16–252). Even though the mean triglyceride levels were clustered at the low end of normal, there were significant differences between groups at the end of the study; cats eating CST had significantly higher triglycerides than cats eating C or CS (*p* < 0.05; Table 2).

### 3.3. Cytokines

Serum levels of cytokines assessed included Flt-3L (FMS-like tyrosine kinase 3), IFN-γ, TNF-α, IL-4, IL-6, IL-8, IL-12, IL-13, IL-18, and MCP-1. The overall effects of the treatments were that CT reduced cytokine concentration while CST increased cytokine concentration. The overall effect was seen in the reduction of concentration in the CT fed cats and an increase in the CST fed cats. Eight out of 10 individual cytokines increased concentration during the feeding period for the CST fed group as shown in Figure 2 (Il-18 and Il-12 were unchanged).

### 3.4. Plasma Metabolomics 

In this study, Metabolon platform detected 563 biochemicals in the plasma samples. Significant changes in biochemicals were identified in all groups when compared to time-matched controls at the end of the study (Table 3). Most of the significant changes in metabolite concentrations in cats eating CT and CS, when compared to cats consuming C, were energy and lipid derived. The carnitine and glycerophosphocholine (GPC) moieties were increased in the CT group while the glycerophosphoethanolamine (GPE) moieties were increased in the CS group. In the CST group, there were some carnitine moieties increased but not carnitine or deoxycarnitine (which were increased in the CT group). Additionally, in the CST group pyruvate and succinate were increased in comparison to the C group (Table 3).

When compared to control at the end of the study, cats eating foods with carnitine (CT and CST) had significantly increased concentrations of tiglycarnitine and isobutyrylcarnitine in plasma (Table 4). The microbiome-tryptophan-related analytes kynurenate and anthranilate were higher in cats eating CS than in cats eating control food. There was a reduction in glycine, betaine, serine, asparagine, glutamine, homoarginine, ornithine, and guanidinoacetate in the CST group as compared to the cats eating C (Table 4). Carnosine supplementation did not impact circulating carnosine concentration (data not shown).

## 4. Discussion

Feeding healthy adult cats either CS or CT resulted in increased lean body mass when compared to either those fed C or CST. This suggests that these are beneficial nutritional interventions for cats both during aging and for those diseases such as renal disease and obesity where lean body mass support is a significant goal of the nutritional management regimen. It has previously been reported that there was a normal loss of body lean during aging [16]. In that study, there was a significant increase in lean body mass during the feeding of a food that contained added L-carnitine. These data suggest that a component of that gain could be attributed to its inclusion. Because (on a dry matter basis) the added L-carnitine in this study was approximately that found in striated muscle, it is possible that this enhanced lean body mass is not a pharmacological response but rather the normal physiological response that might be expected in a species that is an obligatory carnivore. The two physiological conditions where this benefit of L-carnitine might be expected to be most valuable are obesity and renal disease. During obesity, a primary goal of weight loss is the specific loss of body fat while maintaining lean body mass. This has been previously reported [17] with a food containing L-carnitine. These data suggest that a component of that benefit is attributable to L-carnitine. The kidneys are a major site of L-carnitine de novo synthesis, and L-carnitine supplementation is standard therapy for humans with renal disease and dialysis. In the cat, there is a special emphasis on body lean support as loss of lean is associated with reduced quality and length of life during renal disease. A food with increased L-carnitine as well as enhanced amino acids was superior in maintaining body lean compared to a food without these enhancements [18]. It is possible that there could be a specific benefit for each supplementation depending on the metabolic need of the cat. For example, the cats consuming supplemental carnosine by itself had increased lean and reduced adiposity while not changing food intake. This suggests that the metabolic energy requirement for increased body lean was mobilized from body lipid. This is in comparison to the cats consuming supplemental L-carnitine alone, which increased lean while not changing stored lipid or food intake. This suggests that the increased metabolic energy demand for cats consuming L-carnitine alone was met by increased energy efficiency. Further research is needed to isolate if these changes in energy use are beneficial to specific disease states in the cat.

The lack of response in the cats consuming CST is somewhat noteworthy because not only was the positive benefit of the two interventions not additive, the presence of both negated the effect of either alone. This, from a fundamental perspective, is somewhat explained in the measured circulating concentrations. When both dietary interventions appeared together, there was a loss of the increased carnitine and deoxy-carnitine, as well as reductions in betaine, glycine, serine, glutamine, and lysine. These changes suggest that the precursor milieu for protein synthesis may have been negatively influenced by the combination, while this was not true with the inclusion of each alone. If there was a reduction in protein synthesis, it could explain the increased circulating concentration of glucose, pyruvate, and succinate, which may have increased through a lack of metabolic demand.

In general, the immune response to stimulation should be a robust increase in inflammatory cytokines. However, in the absence of stimulation an increased concentration of inflammatory cytokines is considered negative as the cat is likely to experience a low level of inflammation, which is a risk factor for many diseases of aging such as arthritis, cystitis, and nephritis [19,20]. The reduced cytokine concentrations seen with the inclusion of dietary L-carnitine alone are similar to those that were reported in the human [21,22], rats [23], mice [24], and black seabream fish [25]. These data suggest that as in these other species, dietary CT food has a benefit in cats. The overall increase of circulating cytokines with the combination could be valuable in certain disease states to maximize immunological effectiveness. However, it seems reasonable to conclude that in the healthy cat the inclusion of carnitine alone has a benefit in this reduced propensity for inflammation, and the opposite effect was seen with the addition of both L-carnitine and carnosine.

## 5. Conclusions

Dietary L-carnitine or carnosine increased lean mass in cats. Supplementation with L-carnitine reduced circulating cytokine concentrations, while dietary carnosine did not. Feeding a combination of carnitine and carnosine increased circulating concentrations of cytokines and did not enhance lean body mass. These results may be applied in healthy cats to increase lean and in cats fed L-carnitine alone to reduce basal inflammatory cytokines. The study was limited by measurements only in healthy cats, while its optimum application may well be in disease states. Using healthy cats had the advantage of having a low biological variation, allowing for measurements of true difference.

## Figures and Tables

**Table 1:** Effect of dietary carnitine, carnosine, or their combination on body composition of healthy mature adult cats initially and after 169 days of feeding *.

Variable	*Control (C)*	*C±Carnitine*	*C+Carnosine*	*C+Both*
Lean body mass (g) Initial	3712 ± 41	3699 ± 41	3722 ± 39	3702 ± 43
Lean body mass (g) Final	3591 ± 41 ^a^	3805 ± 41 ^b^	3819 ± 39 ^b^	3542 ± 41 ^a^
Fat body mass (g) Initial	1809 ± 72	1794 ± 72	1793 ± 69	1844 ± 76
Fat body mass (g) Final	1998 ± 72 ^a^	1950 ± 72 ^a^	1740 ± 69 ^b^	2097 ± 76 ^a^
Body Weight (g) Initial	5675 ± 65	5678 ± 65	5664 ± 62	5694 ± 65
Body weight (g) Final	5749 ± 65 ^a^	5946 ± 65 ^b^	5712 ± 62 ^a^	5795 ± 69 ^a^
Mean Intake (Kcal/day)	194 ± 17.5	201 ± 16.7	203 ± 16.0	218 ± 17.5

* Least squares means ± standard error of the mean. ^a,b^ Letters denote significant difference (*p* ≤ 0.05) in that row.

**Table 2:** Selected blood chemistry values of healthy mature adult cats initially and after 169 days of feeding *.

	*Control (C)*	*C+Carnitine*	*C+Carnosine*	*C+Both*
Creatinine ^1^ Initial (mg/dL)	1.27 ± 0.06	1.21 ± 0.06	1.37 ± 0.06	1.28 ± 0.06
Creatinine final (mg/dl)	1.20 ± 0.06	1.13 ± 0.06	1.30 ± 0.06	1.10 ± 0.06
Glucose ^2^ initial (mg/dL)	100.4 ± 5.1	107.4 ± 5.1	100.7 ± 4.9	105.4 ± 5.3
Glucose final (mg/dL)	98.0 ^a^ ± 5.1	100.7 ^a^ ± 5.1	103.2 ^a^ ± 4.9	115.0 ^b^ ± 5.3
Triglycerides ^3^ initial (mg/dL)	31.2 ± 3.7	32.6 ± 3.7	27.9 ± 3.5	29.2 ± 3.9
Triglycerides final (mg/dL)	31.3 ^a^ ± 3.7	34.7 ^a,b^ ± 3.6	32.6 ^a^ ± 3.5	44.4 ^b^ ± 3.9
Cholesterol ^4^ initial (mg/dL)	190 ± 15	183 ± 15	167 ± 14	170 ± 15
Cholesterol final (mg/dL)	207 ± 15	213 ± 15	202 ± 14	208 ± 15

* Least squares means ± standard error of the mean. ^a,b^ Letters denote significant difference (*p* < 0.05) in that row. ^1^ Normal range 0.60–2.00. ^2^ Normal range 67.00–157.00. ^3^ Normal range 16.00–252.00. ^4^ Normal range 101.00–267.00.

**Table 3:** Ratio of final/initial concentration of plasma energy and lipid metabolites that were not different initially and were different between treatment and control-fed pets after 169 days of feeding.

Metabolite	*C+Carnitine/C*	*C+Carnosine/C*	*C+Both/C*
pyruvate	0.73	1.05	1.39
succinate	1.08	1.04	1.18
17-methylstearate	1.41	1.15	1.43
malonylcarnitine	1.42	1.35	1.44
butyrylcarnitine (C4)	1.63	1.01	1.36
propionylcarnitine (C3)	1.51	0.96	1.39
hexanoylglycine	0.72	0.73	0.65
hexanoylcarnitine (C6)	1.28	0.87	1
octanoylcarnitine (C8)	1.35	0.93	1.18
suberoylcarnitine (C8-DC)	1.4	1.31	1.5
pimeloylcarnitine/ 3-methyladipoylcarnitine (C7-DC)	1.48	1.16	1.46
cerotoylcarnitine (C26)*	1.52	1.18	1.65
deoxycarnitine	1.32	0.89	1.17
carnitine	1.2	0.95	1.08
linoleoyl ethanolamide	1.27	1.05	1.11
trimethylamine N-oxide	1.34	0.92	1.14
1-linoleoyl-2-linolenoyl-GPC (18:2/18:3)	1.32	1.05	0.94
1-oleoyl-2-linoleoyl-GPC (18:1/18:2)	1.2	1.11	1.05
1-palmitoyl-2-stearoyl-GPC (16:0/18:0)	1.51	1.33	1.34
1,2-dilinoleoyl-GPC (18:2/18:2)	1.3	1.09	1.05
1-palmitoleoyl-GPC (16:1)	1.23	0.97	0.97
1-linolenoyl-GPC (18:3)	1.37	1.02	1.11
1-palmitoyl-GPE (16:0)	0.83	0.8	0.88
1-stearoyl-GPE (18:0)	0.86	0.81	0.88
1-(1-enyl-palmitoyl)-2-arachidonoyl-GPC (P-16:0/20:4)	0.84	0.94	0.76
1-(1-enyl-stearoyl)-2-arachidonoyl-GPE (P-18:0/20:4)	0.82	0.83	0.96
sphinganine-1-phosphate	0.86	0.74	0.72
sphingomyelin (d18:2/16:0, d18:1/16:1)	0.93	0.96	0.87
sphingomyelin (d18:2/24:1, d18:1/24:2)	0.86	0.88	0.8
sphingosine 1-phosphate	0.87	0.8	0.85
sphingomyelin (d18:1/25:0, d19:0/24:1, d20:1/23:0, d19:1/24:0)	1.48	1.02	1.94

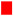
 Means in red are increasing *p* < 0.05. 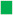 Means in green are decreasing *p* < 0.05.

**Table 4:** Ratio of final/initial concentration of plasma amino acid metabolites that were not different initially and were different between treatment and control-fed pets after 169 days of feeding.

	*C+Carnitine/C*	*C+Carnosine/C*	*C+Both/C*
glycine	0.98	0.98	0.83
betaine	0.81	1.02	0.79
serine	0.98	0.96	0.77
asparagine	0.98	0.94	0.83
glutamine	1	0.95	0.88
N-acetyl-aspartyl-glutamate (NAAG)	0.9	1.1	1.44
S-1-pyrroline-5-carboxylate	0.96	0.94	0.85
histidine	1.03	1.02	0.97
lysine	0.94	0.9	0.81
glutarate (pentanedioate)	1	1.19	1.58
3-(3-hydroxyphenyl)propionate sulfate	1.94	2.47	2.73
3-(3-hydroxyphenyl)propionate	1.63	2.59	2.4
3-(4-hydroxyphenyl)propionate	2.09	4.64	5.51
4-hydroxycinnamate sulfate	3.51	4.15	4.36
3-hydroxyphenylacetate sulfate	2.11	3.05	2.32
kynurenate	1.01	1.31	1.03
anthranilate	1.62	1.62	1.39
picolinate	1.25	1.31	1.3
isovalerylcarnitine (C5)	1.46	0.85	1.2
3-methylglutaconate	1.29	1.07	0.62
tiglylcarnitine (C5:1-DC)	1.47	1.11	1.64
ethylmalonate	1.26	1.17	1.23
valine	0.91	0.92	0.85
isobutyrylcarnitine (C4)	1.86	1.02	1.85
methionine	1.23	1.19	1.24
methionine sulfoxide	1.62	1.28	1.38
2-aminobutyrate	0.75	0.78	0.7
taurine	1.01	0.93	0.83
ornithine	0.93	0.92	0.82
homoarginine	1.03	0.73	0.71
guanidinoacetate	0.76	0.72	0.54
gamma-glutamylmethionine	1.33	1.25	1.39
Phenylacetylcarnitine	2.44	1.37	2.16

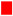
 Means in red are increasing *p* < 0.05. 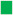 Means in green are decreasing *p* < 0.05.

## Data Availability

Data is contained within the article or Supplementary Materials. The data presented in this study are available by request to the corresponding author.

## Supplementary Materials

The following are available online at https://www.mdpi.com/article/10.3390/biology10040299/s1, Table S1: Food composition of test foods (grams/100 grams).
