# Supplementary material for: Dietary Carnitine and Carnosine Increase Body Lean in Healthy Cats in a Preliminary Study
Source: Biology (Basel). 2021 Apr 5;10(4):299. doi: 10.3390/biology10040299 (PMC8066050; doi:10.3390/biology10040299)
Supplement: Supplementary file 1 [file biology-10-00299-s001.pdf]

biology MDPI

Table S1. Food composition of test foods (grams/100 grams).

Food	Control	Control + L-Carnitine	Control + Carnosine	Control + L-Carnitine and Carnosine
Moisture	6.32	6.47	6.73	6.11
Protein	33.26	33.69	32.75	33.46
Fat	21.18	21.07	19.90	19.23
Atwater Energy [€] (kcal/kg)	4,109	4,102	4,038	
Ash	5.23	5.24	5.04	5.43
Crude fiber	1.30	1.20	1.30	1.30
Calcium	0.90	0.89	0.88	0.95
Phosphorus	0.68	0.62	0.69	0.62
Sodium	0.38	0.37	0.46	0.34
Capric acid [10:0]	0.01	0.02	0.01	0.01
Lauric acid [12:0]	0.02	0.02	0.02	0.02
Myristic acid [14:0]	0.23	0.26	0.22	0.21
Palmitic acid [16:0]	4.22	4.63	4.05	3.90
Palmitoleic acid [16:1]	0.55	0.61	0.52	0.51
Stearic acid [18:0]	1.92	2.12	1.84	1.74
Oleic acid [18:1]	6.89	7.63	6.67	6.34
LA [18:2 (n-6)]	3.66	3.98	3.74	3.55
aLA [18:3 (n-3)]	0.22	0.23	0.22	0.23
ARA [20:4 (n-6)]	0.11	0.11	0.10	0.11
EPA [20:5 (n-3)]	0.10	0.11	0.09	0.09
DHA [22:6 (n-3)]	0.08	0.08	0.07	0.07
Lysine	1.45	1.44	1.29	1.30
Threonine	1.12	1.12	1.11	1.12
Methionine	1.39	1.44	1.38	1.43
Cystine	0.52	0.54	0.53	0.54
Tryptophan	0.32	0.32	0.31	0.32
L-Carnitine (added)	0	0.03	0	0.03
Carnosine (added)	0	0	0.10	0.10

[€] Calculated from analyticals using modified Atwater numbers (kcal/g of 3.5 for protein, 8.5 for fat and 3.5 for nitrogen free extract).
